# Bibliography

**Published:** 1897-11

**Authors:** 


### Bibliography.

LOOSENING TEETH, OR CHRONIC ALVEOLITIS (PYORRHŒA ALVEOLARIS, PHAGEDENIC PERICEMENTITIS, RIGGS'S DISEASE, ETC.). ITS CAUSES, CLINICAL HISTORY, AND TREATMENT. WITH GENERAL DIRECTIONS FOR THE CARE OF THE TEETH. In two volumes. By Dr. Henry S. Nash, New York. Vol. I. New York: Barton F. Welles, 1897.

The author in this volume, upon what he denominates as "chronic alveolitis," has undertaken the serious task of explaining the origin of what is generally understood as pyorrhœa aveolaris. The first volume is now presented to dental readers, and, as stated, is "divided into five parts, for purposes of reference. The first of these will be historical; . . . the second comprises accounts of those of its types which do not concern the dental surgeon; and the third, those which are exclusively within his province.

" Following the notices of the different types of alveolitis, and introductory to the second volume, are some sections upon conditions which are prodromal and predisposing to the class of disorders in mind. These form the fourth and fifth parts."

In the first chapter, " Historical," the author details some of his early difficulties in the treatment of this disease, and freely consulted with Dr. Hamilton in regard to it; and it is interesting to note the following: "The question of its association with gout or rheumatism was one to which we devoted considerable time. I one day showed him some of the deposits called commonly, but, I think, inaccurately, serumal, when he told me to ask each person whose teeth showed its presence if either of these diseases had been experienced by him, or if there had been any history of them in his family. Careful and protracted inquiry demonstrated the fact that while there were cases where the tartar and one of these afflictions were concomitant, in the vast majority of instances they seemed to exist quite independently of each other."

The author, following the lead adopted by many writers upon this subject, seems to have worried over the name usually given the disease,—that of pyorrhœa alveolaris; and of this he "can hardly speak with patience." His efforts to secure a proper name carried him no further than "loosening teeth," although he, in part, adopts Dr. Hamilton's suggestion of "chronic alveolitis." This has the very great merit of simplicity, and would do very well if it were any better than the name heretofore given it and almost universally accepted.

The author seems to be possessed with the idea that this disease has its origin in the alveolar process; for, he says, "the alveolar process has a strong natural tendency to destructive change. . . . Any continued irritation of the gums, like that caused by the pressure of an ill-fitting clasp or plate, or that which they experience from the too vigorous use of a stiff tooth-brush, may occasion its recession." It would seem from this that he entertains the opinion that osteitis is a possibility independent of the periosteum. This, while recognized with some show of reason by writers, is, in the opinion of the reviewer, of doubtful possibility in the alveolar process. In any event, the periosteum and its analogue, the pericementum, and eventually the cement, are destroyed. To confine this disease to the alveolus is an impossibility: hence the name alveolitis is as much of a misnomer as that of pyorrhœa alveolaris. The proof is altogether the other way, for the beginnings of the pathological state are not with the alveolar process, neither with the gingivæ, but with the pericementum, and extending from there to the periosteum.

The author gives this somewhat novel explanation of resorption of the temporary roots: "The roots of the deciduous teeth are removed by phagocytes." Upon what ground this assertion is based does not appear; but it would be interesting to know what gave rise to this new office of the white blood-corpuscle in addition to the many ascribed to it.

The idea of the author as to the origin of pyorrhœa alveolaris or, as he terms it, chronic alveolitis may be condensed in one sentence; for while he does not confine its beginnings to one source, this is regarded as the most important. He ventures, therefore, to call it "idiopathic," although he acknowledges that "no disease is such strictly speaking." And then, further on, says, "I will say briefly that idiopathic alveolitis is entirely of nervous origin."

He describes "the exciting causes of alveolitis" as follows: "A peculiar variety of hypercementosis, which is scarcely ever seen, except with elderly persons, specific disease, atrophy, and scurvy. These form its symptomatic division. Following these are three kinds of metallic poisoning,—lead, mercury, and bismuth. . . . Then there are two varieties which will be termed calcic, as both are induced by tartar; one will be called deep-seated, the other superficial. Next, one will be called eruptive. The eleventh is the most dangerous and mysterious of all. . . . I will . . . call it idiopathic alveolitis."

While the author shares the calcic idea with most writers, he very flatly classifies tartar, presumably salivary tartar, for he does not seem to recognize the serumal as a distinctive formation, or as the cause of alveolitis. It is passing strange that men so differ in observation of plain facts. The writer has never yet seen a case of pyorrhœa under accumulations of tartar, and, theoretically, it should not exist there. Recession of gums, destruction of pericementum and periosteum are necessary sequelæ of continued pressure, but no alveolitis, as he describes it.

The author is undoubtedly correct in objecting to the claim made that it is infectious, for this it certainly is not, whatever else it may be.

The disease is not considered by him as having a microbic origin, for he says, "I know of no reason why they should not become introduced afterwards, however; but, on the other hand, I see none why their agency is essential."

It is impossible to follow the author throughout this book of one hundred and ninety-nine pages, and it must be said of this first volume that, while it is interesting, it does not illumine the subject, and it probably will fail to convince many that his conclusions are, in the main, correct. The story, as he understands it, of chronic alveolitis is told in an entertaining manner; but it would have been decidedly more effective if more condensed. The repetition of ideas adds no strength to the book; in fact, in a measure defeats a clear perception of the author's meaning.

